# Clinical relevance of cell-free DNA in gastrointestinal tract malignancy

**DOI:** 10.18632/oncotarget.13821

**Published:** 2016-12-07

**Authors:** Yuan-Tzu Lan, Ming-Huang Chen, Wen-Liang Fang, Chih-Cheng Hsieh, Chien-Hsing Lin, Fang-Yu Jhang, Shung-Haur Yang, Jen-Kou Lin, Wei-Shone Chen, Jeng-Kai Jiang, Pei-Ching Lin, Shih-Ching Chang

**Affiliations:** ^1^ Division of Colon and Rectal Surgery, Department of Surgery, Taipei Veterans General Hospital, Taipei, Taiwan; ^2^ Department of Surgery, National Yang-Ming University, Taipei, Taiwan; ^3^ Division of Medical Oncology, Department of Oncology, Taipei Veterans General Hospital, Taipei, Taiwan; ^4^ Division of General Surgery, Department of Surgery, Taipei Veterans General Hospital, Taipei, Taiwan; ^5^ Division of Thoracic Surgery, Department of Surgery, Taipei Veterans General Hospital, Taipei, Taiwan; ^6^ School of Medicine, National Yang-Ming University, Taipei, Taiwan; ^7^ Genome Research Center, National Yang-Ming University, Taipei City, Taiwan; ^8^ Department of Clinical Pathology, Yang-Ming Branch, Taipei City Hospital, Taipei, Taiwan

**Keywords:** cfDNA, GI tract cancer, biomarker, carcinoembryonic antigen, response

## Abstract

**Background:**

Cell-free DNA (cfDNA) extracted from blood has become a clinically feasible biomarker in various types of cancer. However, the clinical significance of cfDNA in gastrointestinal (GI) tract cancer among Asian populations requires further investigation.

**Results:**

The median cfDNA copy number was highest in esophageal cancer, followed by colorectal cancer and gastric cancer, which were all significantly higher than those of healthy individuals. The cfDNA levels were higher in GI tract cancer, followed by those in carcinoma *in situ* and then healthy individuals (*P* = 0.019). During the postoperative surveillance, the cfDNA level tended to be more sensitive than the carcinoembryonic antigen level in predicting recurrence. For recurrent gastric cancer, a persistently high cfDNA level and an increasing trend was observed after surgery. For stage IV colorectal cancer, dynamic changes in the cfDNA level were correlated with the responses to chemotherapy and surgery.

**Materials and Methods:**

Blood samples were collected from 95 healthy individuals and from 855 patients diagnosed with GI tract malignancy, including 98 with esophageal cancer, 428 with stomach cancer, 329 with colorectal cancer and 30 with carcinoma *in situ*. The copy numbers of extracted cfDNA were analyzed and compared among the different types of GI cancers.

**Conclusions:**

The cfDNA level can serve as a feasible biomarker for detecting tumors in GI tract cancer. The cfDNA level may play a role in predicting tumor responses to chemotherapy and surgery in colorectal cancer; tumor recurrence should be considered in gastric cancer with a persistently high cfDNA level after surgery.

## INTRODUCTION

Despite advances in diagnostic screenings and therapies, cancer continues to be a major cause of death worldwide [1]. Currently, physicians use image-based Response Evaluation Criteria in Solid Tumors (RECIST) as the gold standard for assessing the initial tumor bulk and defining treatment responses in solid tumors. However, inter- and intraobserver variability and crude categorization limit the use of RECIST [2]. Current blood biomarkers, such as carcinoembryonic antigen (CEA), in colorectal cancer could improve the use of image-based assessments, but the sensitivity and specificity of these markers are moderate [3–5].

With the progression of molecular techniques, the detection of cell-free DNA (cfDNA) in blood has become clinically feasible [6]. Obviously higher cfDNA levels in the blood of cancer patients have rendered cfDNA analysis a novel method for assessing patient diagnosis, prognosis, and follow-up status [6]. In addition to quantitative differences in the cfDNA levels between cancer patients and healthy individuals, cfDNA molecules have been found to exhibit genetic or epigenetic alterations, including mutations and differences in methylation and genomic copy numbers compared with those from tumor cells [7, 8].

A previous report has demonstrated that tumor-derived cfDNA is highly fragmented and mostly smaller than 100 bp in size [9]. The level of cfDNA is expected to be associated with tumor volume; in addition, some studies have shown that cfDNA might also originate from peripheral cells and tissue surrounding tumors, thereby reflecting the complex microenvironments within cancer patients [10].

Previously, we demonstrated that the outcomes of colorectal cancer patients with low pretreatment cfDNA levels were significantly better than those of patients with high cfDNA levels [11]. In gastric cancer, patients with high cfDNA levels were more likely to experience peritoneal recurrence and exhibited significantly lower 5-year overall survival rates than patients with low cfDNA levels [12].

Consequently, cfDNA might be of clinical value in GI tract cancer. The aim of the present study was to evaluate the cfDNA levels in different GI tract cancer types and analyze the dynamic changes in cfDNA levels before and after treatment in colorectal cancer patients.

## RESULTS

This study enrolled 980 patients, including 98 with esophageal cancer, 428 with stomach cancer, 329 with colorectal cancer, 30 with colorectal carcinoma *in situ*, and 95 healthy individuals. The demographic characteristics of the GI tract cancer patients and healthy individuals are shown in Table 1. The median age of the healthy individuals was 54.2, with a male-to-female ratio of 66:29. For cfDNA extraction and quantification, pooled standard plasma samples and serially diluted standard DNA were used to measure the cfDNA level of each individual. The TaqMan assay *R*^2^-value of the serially diluted standard DNA (0.001, 0.01, 0.1, 1, 10, 100 ng) was 0.9826 ± 0.02, and the batch-effect factors ranged from 0.91~1.16. The mean cfDNA level in the healthy individuals was 613 ± 888 copies/mL (median: 167; range 0–4,156) and was similar in males and females, at 529 ± 828 and 804 ± 1,001 copies/mL, respectively (*P* = 0.166). There was no significant difference between patients younger and older than 65 years old.

**Table 1:** Demographic characteristics of GI tract cancer patients and healthy individuals

	Healthy individuals *n* = 95	Esophageal cancer *n* = 98	Stomach cancer *n* = 428	Colorectal cancer *n* = 329
Age (y/o)	54.2 ± 15.5	60.1 ± 12.0	65.7 ± 13.3	69.2 ± 13.1
Gender				
Male	66 (69.5)	85 (86.7)	307 (71.7)	181 (55.2)
Female	29 (30.5)	13 (13.3)	121 (28.3)	148 (44.8)
TNM stage				
I		15 (15.3)	81 (18.9)	60 (18.2)
II		20 (20.4)	103 (24.1)	115 (35.0)
III		63 (64.3)	207 (48.4)	80 (24.3)
IV		0 (0)	37 (8.6)	74 (22.5)
cfDNA level (copies/mL)				
Mean	613 ± 888	29968 ± 35455	11514 ± 27817	17774 ± 86783
Median	167	13494	4174	6192
Range	0–4156	1126–161170	0–380749	910–1534219

In Table 2, the cfDNA levels were significantly higher in GI tract malignancy, followed by those in carcinoma *in situ* and then healthy individuals (16,038 ± 58,787 vs. 3,249 ± 1,336 vs. 613 ± 888, *P* = 0.019).

**Table 2:** cfDNA levels among GI tract cancer, carcinoma *in situ*, and healthy individuals

	Healthy individuals *n* = 95	Carcinoma *in situ* *n* = 30	GI tract cancer *n* = 855	*P* value
cfDNA level (copies/mL)				0.019
Mean	613 ± 888	3249 ± 1336	16038 ± 58787	
Median	167	2848	5497	
Range	0–4156	1057–5579	0–1534219	

### cfDNA level in different types and stages of GI tract malignancies

**Figure 1:**
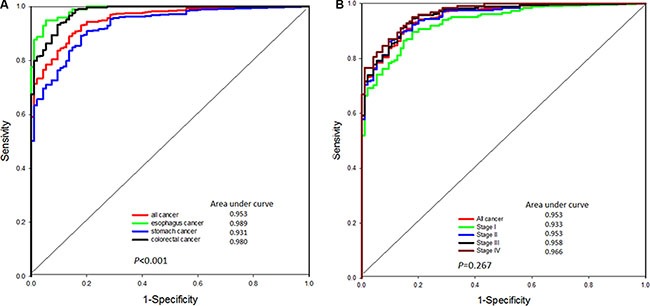
(**A**) The ROC curve of cfDNA levels in healthy individuals and patients with different types of GI tract cancer. (**B**) The ROC curve of cfDNA levels in healthy individuals and patients with different stages of GI tract cancer.

The median cfDNA level was highest in esophageal cancer patients (13,494; range: 1,126–161,170) followed by those of colorectal cancer patients (6,192; range: 910–1,534,219) and stomach cancer patients (4,174; range: 0–380,749). The cfDNA levels in patients with any type of cancer were significantly higher than those in the healthy individuals. To estimate the diagnostic value of the cfDNA tested, the data from each group of cancer patients was compared with those of the healthy controls (Figure 1A). The Receiver operating characteristic (ROC) analyses revealed an area under the curve (AUC) of 0.989 (95% CI: 0.979–0.998) for esophageal cancer, 0.931 (95% CI: 0. 907–0.955) for stomach cancer, 0.980 (95% CI: 0.968–0.992) for colorectal cancer, and 0.952 (95% CI: 0.935–0.970) for all cancer types compared with the controls. For differential diagnosis, an optimal cut-off value of 2,700 copies/mL was selected to distinguish cases of cancer from normal cases with a specificity of 95.8% and a sensitivity of 75.8%. The sensitivity was 75.9%, 68.9%, and 82.7% for esophageal, stomach, and colorectal cancer, respectively (Figure 1A).

For the severity of disease in these GI tract malignancies, the ROC analyses showed an AUC of 0.918 (95% CI: 0.887–0.949) in stage I disease, 0.935 (95% CI: 0.915–0.964) in stage II disease, 0.945 (95% CI: 0.924–0.967) in stage III disease and 0.956 (95% CI: 0.934–0.979) in stage IV disease. The sensitivity of stage I, II, III and IV disease was 70.6%, 75.6%, 77.5% and 80.7%, respectively, with the cut-off value of 2,700 copies/mL. (Figure 1B)

### Dynamic changes in cfDNA levels before and after tumor resection

Postoperative blood samples were available from some patients of our gastric and colorectal cancer patients. Dynamic changes in cfDNA levels were studied in these cases but not in cases of esophageal cancer.

### Gastric cancer

Before 2008, adjuvant chemotherapy or radiotherapy was not routinely performed after curative surgery in our gastric cancer patients only when tumor recurrence was diagnosed. Other adjuvant therapies, such as TS-1, have been prescribed for stage II or stage III patients after curative surgery in our hospital since 2008 due to the demonstrated benefits for survival [13].

**Figure 2:**
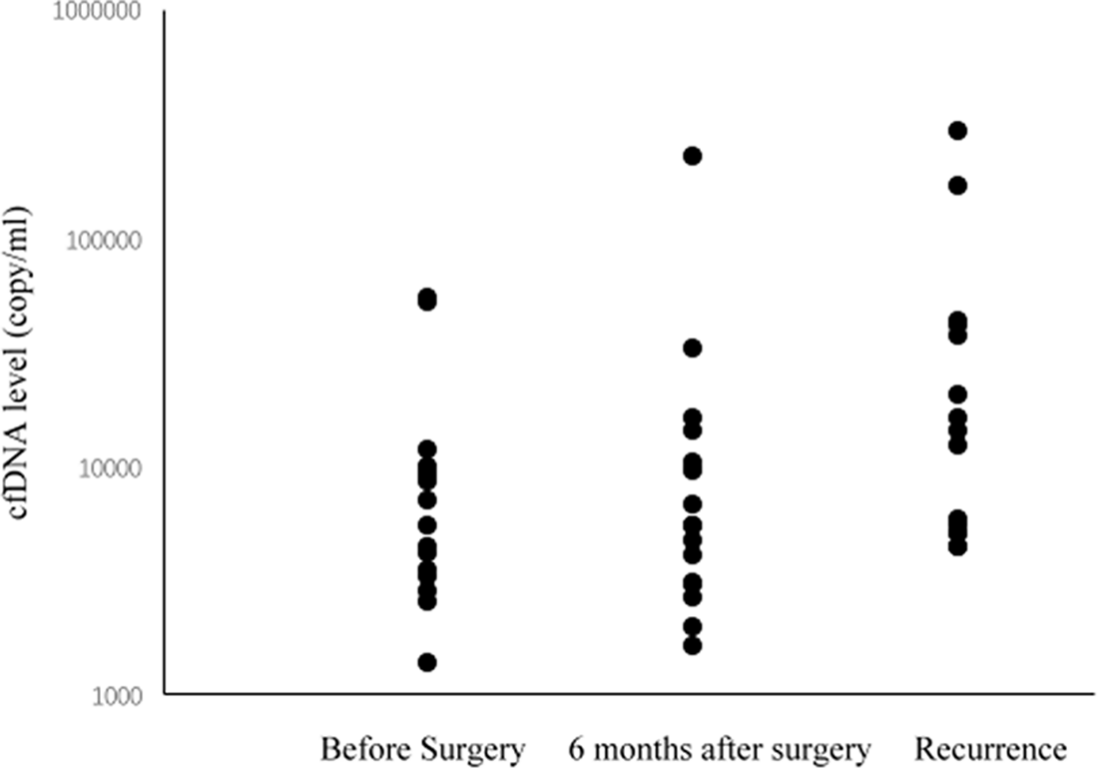
cfDNA levels before surgery, 6 months after surgery, and at the time of recurrence in gastric cancer patients

Pre- and postoperative cfDNA levels (six months after surgery and at the time of recurrence) were measured in 18 gastric cancer patients who did not receive adjuvant chemotherapy. All 18 patients had high pre- and postoperative cfDNA levels at six months after surgery and at the time of recurrence. As shown in Figure 2, persistently high and increasing cfDNA levels were observed after surgery in gastric cancer patients with tumor recurrence.

### Colorectal cancer

**Figure 3:**
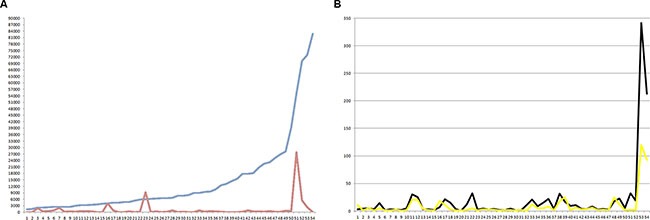
(**A**) Dynamic changes in cfDNA levels before (blue line) and after (red line) colorectal cancer tumor resection. (**B**) Dynamic changes in serum CEA levels before (black line) and after (yellow line) colorectal cancer tumor resection.

Pre- and postoperative cfDNA levels were measured in 54 colorectal patients receiving curative tumor resection. Of these patients, two patients (3.7%) had higher postoperative cfDNA levels than preoperative cfDNA levels. The cfDNA level of four patients (7.4%) was higher than 2,700 copies/mL, which was set as the cut-off value of normal limits by our previous results (Figure 3A). The dynamic changes in the CEA level showed that 3 (5.6%) cases had higher postoperative CEA levels than preoperative CEA levels, and the CEA level did not return to the normal level of 5 ng/ml in 16 (29.6%) cases (Figure 3B). Among the four cases in which the cfDNA level did not return to normal, liver metastasis occurred in two cases within two years after the operation. In four of the sixteen cases (25%) with a high postoperative CEA level, tumor recurrence occurred in the liver, lungs and peritoneum.

### Dynamic changes in cfDNA levels in metastatic colorectal cancer patients receiving chemotherapy

**Figure 4:**
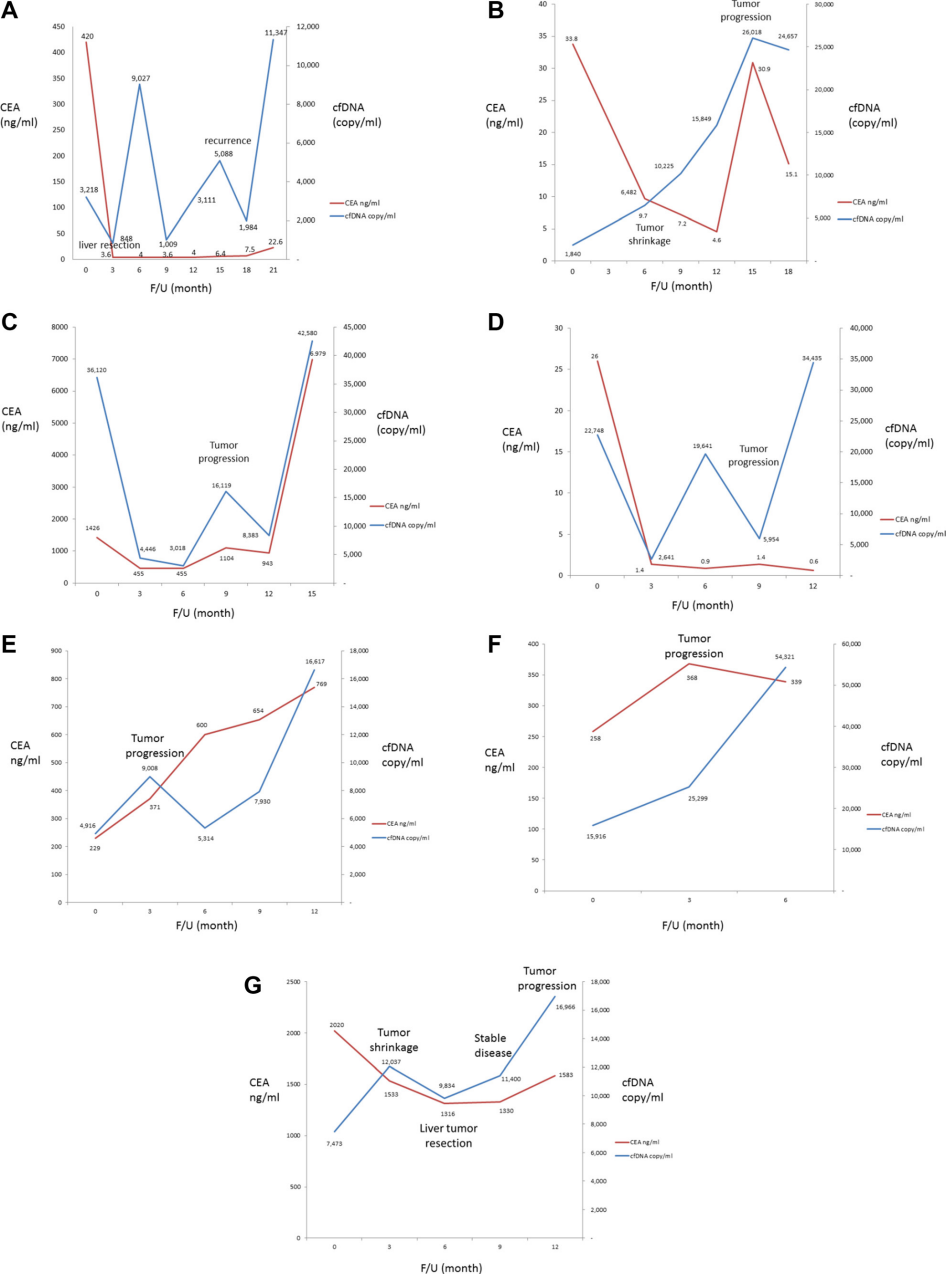
Dynamic changes in cfDNA levels in seven metastatic colorectal cancer patients receiving chemotherapy

Among the 10 metastatic colorectal patients, 4 patients had a follow-up period longer than 18 months. The dynamic change in the cfDNA level are shown in Figure 4.

The first patient (Figure 4A) initially received bevacizumab plus FOLFIRI for two months and showed a partial response. One month later, primary tumor and metastatic liver tumor resections were performed. The patient maintained stable disease for 15 months with subsequent disease progression; another liver resection was performed at that time. As shown in Figure 4A, the levels of both CEA and cfDNA decreased after the initial systemic chemotherapy and were the lowest just after tumor resection. The CEA level remained stable during the period of clinically stable disease; however, two peaks were observed in the level of cfDNA. Both the cfDNA levels (1,984 vs. 11,347 copies/mL) and CEA levels (7.5 vs. 22.6 ng/mL) increased with disease progression.

The second patient (Figure 4B) had unresectable metastatic disease and initially received systemic chemotherapy with bevacizumab plus FOLFIRI followed by FOLFOX. The patient showed a partial response 3 months later and exhibited stable disease 9 months after treatment, as determined by imaged-based RECIST criteria. However, the patient showed disease progression 12 months after the initial treatment. As shown in Figure 4B, the CEA level was the lowest at 9 months after treatment, the same time at which stable disease was reached, as mentioned above. In contrast, the cfDNA level continuously increased, even during the period of clinically stable disease

The third patient (Figure 4C) initially received treatment with bevacizumab plus FOLFORI. The patient showed a partial response 3 months later, and both the serum CEA level and cfDNA levels dropped. The patient exhibited disease progression 9 months after treatment. Both the serum CEA and cfDNA levels were elevated at that time. The chemotherapy regimen was changed to aflibercept plus FOLFOX, and the patient subsequently showed stable disease; both the serum CEA and cfDNA levels dropped again. However, the disease progressed 6 months after the chemotherapy regimen was changed, and the serum CEA level and cfDNA levels increased again. The treatment regimen was then changed to regorafenib. The patient died within 2 months after disease progression.

The fourth patient (Figure 4D) also received bevacizumab and FOLFIRI after diagnosis and exhibited a partial response after 2 months of treatment. The patient received metastatic liver tumor resection at 5 months after treatment. The patient showed stable disease without tumor recurrence for 11 months, followed by disease progression. As shown in Figure 4D, the levels of both CEA and cfDNA decreased after 3 months of treatment. The cfDNA level dramatically increased after disease progression; however, the CEA level remained within normal limits even with disease progression.

The fifth patient (Figure 4E) received palliative primary tumor resection initially followed by systemic chemotherapy with bevacizumab plus FOLFIRI and exhibited stable disease 3 months later. The patient had progressive lung metastasis at 9 months after diagnosis. The regimen was shifted to bevacizumabe plus XELOX, and the patient exhibited stable disease again 3 months later. The patient received lung metastasectomy at 15 months after diagnosis and showed disease progression 2 months later. As shown in Figure 4E, the cfDNA level was increased at 6 months after diagnosis, which was before clinical disease progression was observed.

The sixth patient (Figure 4F) received systemic chemotherapy with bevacizumab plus FOLFIRI and exhibited disease progression 3 months later; both the serum CEA and cfDNA levels were elevated. The regimen was then shifted to FOLFOX, and the cfDNA level decreased, but the serum CEA level increased. Subsequently, the patient exhibited disease progression, and both the serum CEA level and cfDNA levels increased. Finally, the patient died of cancer 13 months after the initial treatment.

The seventh patient (Figure 4G) received treatment with bevacizumab plus FOLFORI. The patient exhibited a partial response with liver tumor shrinkage 3 months later; the serum CEA level decreased, but the cfDNA level increased. The patient underwent liver resection 3 months after the partial response and both the serum CEA level and cfDNA levels dropped. The patient remained in a condition of stable disease for 6 months and then exhibited disease progression; during this period, the cfDNA level was more prominently elevated than the serum CEA level.

## DISCUSSION

As confirmed by ROC curve analyses, our results showed that the cfDNA levels in patients with different types of GI tract cancer were significantly higher than those of the healthy individuals and could be used to distinguish between the healthy individuals and each cancer stage. There was a trend of elevated cfDNA levels in gastric cancer patients with tumor recurrence after surgery. Dynamic changes in the cfDNA levels were also observed in colorectal cancer patients receiving curative surgical resection and in metastatic colorectal patients receiving chemotherapy and surgery.

Our results demonstrated that the cfDNA level was significantly higher in patients with GI tract cancer than in healthy individuals. The cfDNA level could be a useful biomarker for distinguishing cancer patients from healthy individuals. Furthermore, the ROC curves also confirmed that the cfDNA level could be a useful biomarker to distinguish normal GI tract tissue from that of each stage of GI tract cancer. As a result, the cfDNA level has potential as a biomarker in GI cancer detection.

Elevation of cfDNA level has been reported in benign disease. For examples, patients with endometriosis have been reported to have significantly higher plasma concentrations of cfDNA than those without endometriosis [14]. In breast lesions, cfDNA levels were higher in cases of benign and malignant breast lesions compared with healthy controls, and the cfDNA levels were higher in patients with malignant breast disease than in either patients with benign disease or healthy controls [15]. Interestingly, our results demonstrated that cfDNA levels were significantly higher in GI malignancies, followed by carcinoma *in situ* and then healthy controls. These findings suggest that the cfDNA level is correlated with the progression of carcinogenesis in GI tract disease.

The results showed that the median cfDNA level was highest in esophageal cancer, followed by stomach and colorectal cancer. This difference might be due to differences in tumor stage at the time of diagnosis and differences in the inherent nature of these tumor types. The significance of different cfDNA levels in cases of cancer affecting different organs requires further evaluation.

Plasma cfDNA levels have been reported to be capable of predicting the efficacy of targeted therapy in patients with metastatic renal cell carcinoma [16]. cfDNA could be not only a diagnostic marker but also a marker of prognostic value in ovarian cancer [17, 18]. cfDNA has clinical utility of for detecting and monitoring breast cancer, and cfDNA mutation profiling can also serve as a tool for identifying biomarkers in patients receiving tamoxifen [19, 20, 21]. In GI tract malignancies, cfDNA levels could serve as a diagnostic and prognostic marker as well as a tool for tumor monitoring and the early detection of recurrence in colorectal cancer patients who have received surgery or chemotherapy [22, 23]. In the present study, dynamic changes were observed in colorectal cancer not only after curative surgical resection but also in patients with metastatic disease receiving chemotherapy and surgery. As shown in Figure 3, in patients with disease progression, elevated cfDNA levels could be detected earlier than elevated serum CEA levels. It seems that the cfDNA level might be more sensitive than the CEA level with respect to tumor progression in metastatic colorectal cancer. Measuring the cfDNA level is useful for screening and monitoring of GI tumors, and the post- chemotherapy elevation in cfDNA could result not only from cancer relapse but also from chemotherapy due to the destruction of malignant cells. Elevated levels of ctDNA were reported in breast cancer patients and possibly in colorectal cancer patients after chemotherapy due to the destruction of cancer cells [24, 25]. For example, as shown in Figure 4G (the 7th case of stage IV colorectal cancer), although the metastatic liver tumor decreased in size and the serum CEA level dropped after chemotherapy, the cfDNA level still increased; the cfDNA level decreased later after liver tumor resection. Hence, differential diagnosis should be applied and should incorporate the post-treatment time point at which the cfDNA level is determined.

Our results demonstrated a high cfDNA level before surgery and a trend of cfDNA elevation after surgery in eighteen gastric cancer patients with tumor recurrence. Among them, preoperative serum CEA levels were elevated in only four (22.2%) patients, while all patients had high preoperative cfDNA levels. Physicians should pay attention to the high possibility of gastric cancer recurrence in patients with high cfDNA levels both pre- and postoperatively, even in cases with higher cfDNA levels after surgery.

Carcinogenesis and tumor progression are complex and progressive processes that are associated with numerous genetic and epigenetic alterations, some of which can also be detected in cfDNA, which may be more specific and accurate than protein biomarkers and have potential as blood biomarkers for cancer. Histological evaluations of blood samples as well as tumor tissues obtained from biopsies are the gold standards for diagnosing cancer; however, most studies typically only conduct these evaluations once. Metastatic and primary tumors from the same patient can vary at the genomic, epigenomic and transcriptomic levels; thus, assays that allow the repetitive monitoring of these events using blood samples would facilitate the assessment of cancer progression in patients from whom tumor tissue is not available [26–28]. cfDNA might have the potential to replace other blood biomarkers for predicting the prognosis of cancer patients in the future. As the individual genomic profiles of a patient tumors become more readily available, cfDNA assays can be better applied in personalized medicine and for monitoring treatment efficacy.

## MATERIALS AND METHODS

### Patients and sample collection

Serum samples were collected from 98 esophageal cancer patients, 428 gastric cancer patients, 329 colorectal cancer patients, 30 colorectal carcinoma *in situ* patients, and 95 healthy individuals. The blood samples were obtained from the biobank at the Taipei Veterans General Hospital between 2005 and 2010. All patients enrolled in the present study had documented preoperative cfDNA levels. The 95 healthy individuals were selected from volunteer blood donors who had no history of malignant disease. All GI tract cancer patients had preoperative cfDNA level data. Among the 329 colorectal cancer patients, 54 patients had both pre- and postoperative cfDNA levels documented. Among the 428 gastric cancer patients, 18 patients had cfDNA levels measured before surgery, 6 months after surgery, and at the time of diagnosis. Informed consent was obtained from all volunteers before the blood was drawn. All samples were anonymous and obtained with written informed consent. The study was approved by the Institutional Review Board of the Taipei Veterans General Hospital.

### Serum DNA extraction

DNA was extracted from plasma using a QIAamp DNA Tissue Kit and a MinElute Virus Kit (Qiagen, Valencia, CA) according to the manufacturer’s instructions. DNA quality and quantity were confirmed using a NanoDrop 1000 Spectrophotometer (Thermo Scientific).

### Reference cfDNA values

The concentration of cfDNA in each sample was calculated according to a standard curve. A ROC curve was created to select the Youden’s index (Youden’s index = sensitivity + specificity − 1), and the highest sensitivity and specificity were selected as the cut-off values. Values greater than or equal to the cut-off values were considered positive, and smaller values were considered negative.

### Quantification of the circulating DNA copy number

A TaqMan quantitative polymerase chain reaction (qPCR) assay of the housekeeping gene cyclophilin, which is not known to be correlated with cancer, was used to quantify the cfDNA copy numbers in the plasma samples [11]. qPCR was performed using TaKaRa Ex Master Mix (TaKaRa Bio, Japan). Serially diluted standard DNA was used to generate a standard curve. The results were expressed as the threshold cycle (Ct), i.e., the cycle number at which the PCR product crossed the threshold of detection. To reduce the batch effect, we prepared a large volume tube of pre-mixed plasma samples (20 ml pooled from multiple samples) and prepared small aliquots of the pooled samples in standard tubes (1 ml) for storage at −80°C. When performing cfDNA extraction and qPCR experiment, we used plasma samples from clinical individuals and the pre-mix standard tube. The cfDNA copy number in each patient was measured according to the Ct value and the standard curve from serially-diluted DNA (0.001, 0.01, 0.1, 1, 10, 100 ng). The results of standard tubes between different batches were used to calculate the batch-effect factor for adjusting the copies/mL value in the following analyses. The ith batch-effect factor was calculated based on the ith pre-mix standard plasma cfDNA level/1st pre-mix standard plasma cfDNA level. Subsequently, the cfDNA copy number was normalized according to the plasma input volume and ith batch-effect factor, and was expressed in copies/mL. The plasma DNA samples of another 95 healthy individuals were used to create a ROC curve, and the optimal cut-off value of cfDNA for distinguishing healthy individuals from GI tract cancer patients was 2,700 copies/mL (Figure 1).

### Statistical analysis

Statistical analyses were performed using SPSS (version 18.0 for Windows, SPSS, Chicago, IL, USA). A ROC curve was generated using STATA software to identify the optimal cut-off value for stratifying patients at a high risk of gastric cancer. In this ROC curve, the point with the maximum sensitivity and specificity was selected as the optimal cut-off value.

## CONCLUSIONS

cfDNA may serve as a useful biomarker in cancer detection, with sufficient sensitivity for monitoring GI cancer patients responses before and after treatment.
